# A mosaic tetracycline resistance gene *tet*(S/M) detected in an MDR pneumococcal CC230 lineage that underwent capsular switching in South Africa

**DOI:** 10.1093/jac/dkz477

**Published:** 2019-12-02

**Authors:** Stephanie W Lo, Rebecca A Gladstone, Andries J van Tonder, Mignon Du Plessis, Jennifer E Cornick, Paulina A Hawkins, Shabir A Madhi, Susan A Nzenze, Rama Kandasamy, K L Ravikumar, Naima Elmdaghri, Brenda Kwambana-Adams, Samanta Cristine Grassi Almeida, Anna Skoczynska, Ekaterina Egorova, Leonid Titov, Samir K Saha, Metka Paragi, Dean B Everett, Martin Antonio, Keith P Klugman, Yuan Li, Benjamin J Metcalf, Bernard Beall, Lesley McGee, Robert F Breiman, Stephen D Bentley, Anne von Gottberg, Global Pneumococcal Sequencing Consortium, Abdullah W Brooks, Alejandra Corso, Alexander Davydov, Alison Maguire, Andrew J Pollard, Anmol Kiran, Anna Skoczynska, Benild Moiane, Betuel Sigauque, David Aanensen, Deborah Lehmann, Diego Faccone, Ebenezer Foster-Nyarko, Ebrima Bojang, Elena Voropaeva, Eric Sampane-Donkor, Ewa Sadowy, Geetha Nagaraj, Godfrey Bigogo, Helio Mucavele, Houria Belabbès, Idrissa Diawara, Jennifer Moïsi, Jennifer Verani, Jeremy Keenan, Jyothish N Nair Thulasee Bhai, Kedibone M Ndlangisa, Khalid Zerouali, Linda De Gouveia, Maaike Alaerts, Maria-Cristina de Cunto Brandileone, Margaret Ip, Md Hasanuzzaman, Metka Paragi, Mushal Ali, Nicholas Croucher, Nicole Wolter, Noga Givon-Lavi, Özgen Köseoglu Eser, Pak Leung Ho, Patrick E Akpaka, Paul Turner, Paula Gagetti, Peggy-Estelle Tientcheu, Philip E Carter, Pierra Law, Rachel Benisty, Rafal Mostowy, Rebecca Ford, Rebecca Henderson, Roly Malaker, Ron Dagan, Sadia Shakoor, Sanjay Doiphode, Sanjay Doiphode, Shamala Devi Sekaran, Somporn Srifuengfung, Shamala Devi Sekaran, Somporn Srifuengfung, Stephen Obaro, Stuart C Clarke, Tamara Kastrin, Theresa J Ochoa, Waleria Hryniewicz, Veeraraghavan Balaji, Yulia Urban

**Affiliations:** 1 Parasites and Microbes Programme, The Wellcome Sanger Institute, Wellcome Genome Campus, Hinxton, Cambridge CB10 1SA, UK; 2 Centre for Respiratory Disease and Meningitis, National Institute for Communicable Diseases, Johannesburg, South Africa; 3 School of Pathology, University of the Witwatersrand, Johannesburg, South Africa; 4 Malawi Liverpool Wellcome Trust Clinical Research Programme, PO Box 30096, Blantyre, Malawi; 5 Institute of Infection & Global Health, University of Liverpool, Liverpool L69 7BE, UK; 6 Hubert Department of Global Health, Rollins School of Public Health, Emory University, Atlanta, GA 30322, USA; 7 Medical Research Council: Respiratory and Meningeal Pathogens Research Unit, University of the Witwatersrand, Johannesburg, South Africa; 8 Department of Science and Technology/National Research Foundation: Vaccine Preventable Diseases, University of the Witwatersrand, Johannesburg, South Africa; 9 Oxford Vaccine Group, Department of Paediatrics, University of Oxford, and the NIHR Oxford Biomedical Research Centre, Oxford OX3 9DU, UK; 10 Department of Microbiology, Kempegowda Institute of Medical Sciences Hospital & Research Centre, Bangalore, India; 11 Department of Microbiology, Faculty of Medicine and Pharmacy, B.P. 9154, Hassan II University of Casablanca, Casablanca, Morocco; 12 Bacteriology-Virology and Hospital Hygiene Laboratory, University Hospital Centre Ibn Rochd, Casablanca, Morocco; 13 NIHR Global Health Research Unit on Mucosal Pathogens, Division of Infection and Immunity, University College London, London, UK; 14 WHO Collaborating Centre for New Vaccines Surveillance, Medical Research Council Unit, The Gambia at The London School of Hygiene and Tropical Medicine, Fajara, The Gambia; 15 National Laboratory for Meningitis and Pneumococcal Infections, Center of Bacteriology, Institute Adolfo Lutz (IAL), São Paulo, Brazil; 16 Department of Epidemiology and Clinical Microbiology, National Medicines Institute, Warsaw, Poland; 17 Laboratory of Clinical Microbiology and Biotechnology, Moscow Research Institute for Epidemiology and Microbiology, Moscow, Russian Federation; 18 Laboratory of Clinical and Experimental Microbiology, The Republican Research and Practical Center for Epidemiology and Microbiology, Minsk, Belarus; 19 Department of Microbiology, Dhaka Shishu (Children’s) Hospital, Child Health Research Foundation, Dhaka, Bangladesh; 20 Department for Public Health Microbiology, National Laboratory of Health, Environment and Food, Maribor, Slovenia; 21 University of Edinburgh, The Queens Medical Research Institute, Edinburgh EH16 4TJ, UK; 22 Respiratory Diseases Branch, Centers for Disease Control and Prevention, Atlanta, GA 30333, USA; 23 Emory Global Health Institute, Emory University, Atlanta, GA 30322, USA

## Abstract

**Objectives:**

We reported *tet*(S/M) in *Streptococcus pneumoniae* and investigated its temporal spread in relation to nationwide clinical interventions.

**Methods:**

We whole-genome sequenced 12 254 pneumococcal isolates from 29 countries on an Illumina HiSeq sequencer. Serotype, multilocus ST and antibiotic resistance were inferred from genomes. An SNP tree was built using Gubbins. Temporal spread was reconstructed using a birth–death model.

**Results:**

We identified *tet*(S/M) in 131 pneumococcal isolates and none carried other known *tet* genes. Tetracycline susceptibility testing results were available for 121 *tet*(S/M)-positive isolates and all were resistant. A majority (74%) of *tet*(S/M)-positive isolates were from South Africa and caused invasive diseases among young children (59% HIV positive, where HIV status was available). All but two *tet*(S/M)-positive isolates belonged to clonal complex (CC) 230. A global phylogeny of CC230 (*n*=389) revealed that *tet*(S/M)-positive isolates formed a sublineage predicted to exhibit resistance to penicillin, co-trimoxazole, erythromycin and tetracycline. The birth–death model detected an unrecognized outbreak of this sublineage in South Africa between 2000 and 2004 with expected secondary infections (effective reproductive number, R) of ∼2.5. R declined to ∼1.0 in 2005 and <1.0 in 2012. The declining epidemic could be related to improved access to ART in 2004 and introduction of pneumococcal conjugate vaccine (PCV) in 2009. Capsular switching from vaccine serotype 14 to non-vaccine serotype 23A was observed within the sublineage.

**Conclusions:**

The prevalence of *tet*(S/M) in pneumococci was low and its dissemination was due to an unrecognized outbreak of CC230 in South Africa. Capsular switching in this MDR sublineage highlighted its potential to continue to cause disease in the post-PCV13 era.

## Introduction

*Streptococcus pneumoniae* is a major bacterial cause of disease in young children. Despite the success of pneumococcal conjugate vaccines (PCVs), invasive pneumococcal disease (IPD) remains an important health priority owing to increasing disease incidence caused by pneumococci expressing non-vaccine serotypes, a phenomenon known as serotype replacement.^1^ Serotype replacement could be mediated by capsular switching, in which a *cps* locus encoding vaccine-type (VT) capsule is replaced by a *cps* locus encoding non-vaccine-type (NVT) capsule through homologous recombination.^2^ Capsular switching within MDR lineages, especially those recognized by the Pneumococcal Molecular Epidemiology Network (PMEN; http://spneumoniae.mlst.net/pmen/pmen.asp), is of increasing concern, as these expansions can reduce overall vaccine effectiveness in preventing IPD and temper the reduction in antimicrobial-resistant pneumococcal infections associated with introduction of PCVs.^3^ The persistence of the MDR lineage ST156 (Spain9^V^-3, PMEN3) in the USA following the introduction of PCV13 provides a clear example of a historically successful lineage that underwent a capsular switch from VT (serotype 9V, 14 and 19A) to NVT (serotype 35B) and continued to cause IPD in the post-vaccine era.^3–5^

Resistance to tetracycline has been frequently observed in *S. pneumoniae.*^6^ The genetic basis was shown to be *tet*(M), and less commonly *tet*(O), which encode for a ribosomal protection protein that prevents tetracycline binding to the bacterial 30S ribosome subunit.^6^^,^^7^ Eleven other classes of ribosomal protection proteins such as *tet*(S) and 12 mosaic structures of *tet* genes such as *tet*(S/M) have not been found previously in pneumococci (http://faculty.washington.edu/marilynr/). *tet*(S), originally discovered in *Listeria monocytogenes* strain BM4210,^8^ has occasionally been found in a variety of streptococci, including *Streptococcus suis* (NCBI accession number KX077886),^9^*Streptococcus infantis* (NCBI accession number JX275965) and *Streptococcus dysgalactiae* (NCBI accession number EF682210)^10^ and is associated with a transposase-containing element IS*1216*, which potentially mediates chromosomal rearrangement. The mosaic *tet*(S/M) has been observed on a Tn*916* element in *Streptococcus intermedius*^11^ and an IS*1216* composite in *Streptococcus bovis.*^12^ Using a dataset of 12 254 pneumococcal genomes from the Global Pneumococcal Sequencing (GPS) project (https://www.pneumogen.net/gps/), we identified a novel genetic basis for tetracycline resistance in *S. pneumoniae*, *tet*(S/M), and characterized its genetic background in relation to nationwide clinical interventions.

## Materials and methods

### Isolate collection

In the GPS project, each participating country retrospectively and randomly selected pneumococcal disease isolates collected via laboratory-based surveillance and carriage isolates via cohort studies using the following criteria: ∼50% of isolates from children ≤2 years old, 25% from children 3–5 years old and 25% from individuals >5 years old. By May 2017 (last access to the GPS database for this study), 12 254 isolates, representing 29 countries, in Africa (65%), North America (14%), Asia (9%), South America (8%) and Europe (4%), were sequenced, passed quality control and included in this study. The collection spanned 26 years between 1991 and 2016 and included both pneumococcal carriage (*n*=4863) and disease isolates (*n*=7391). We compiled the metadata including age, year of collection, sample source, HIV status and phenotypic antimicrobial susceptibility testing results, where available, from each participating site. In children <18 months of age, HIV status was confirmed by PCR assay. Tetracycline resistance phenotype of *tet*(S/M)-positive isolates was confirmed by either microbroth dilution or Etest. MIC results were interpreted according to CLSI M100-S24.^13^ When MIC was analysed as ‘>X’, MIC was approximated as value 2X for median and IQR calculations.

### Genome sequencing and analyses

The pneumococcal isolates were whole-genome sequenced on an Illumina HiSeq platform and raw data were deposited in the European Nucleotide Archive (ENA) (Supplementary metadata, available as Supplementary data at *JAC* Online). We inferred serotype, multilocus ST and resistance profile for penicillin, chloramphenicol, co-trimoxazole, erythromycin and tetracycline from the genomic data, as previously described.^14^ The *tet*(S/M) gene was identified with a *tet*(S/M) reference sequence (NCBI accession number AY534326) using ARIBA^15^ and the promoter region was examined manually by comparing with the reference sequences of *tet*(M) (NCBI accession number M85225) and *tet*(S) (NCBI accession number FN555436). We detected the presence of other *tet* genes in *tet*(S/M)-positive isolates by BLASTing their assemblies against a list of 13 *tet* genes encoding ribosomal protection proteins (Table 1).

**Table 1.** List of *tet* genes encoding ribosomal protection proteins for BLAST analysis

Gene(s)	NCBI accession number
*tet*(M)	MH283017
*tet*(O)	Y07780
*tetA*(P) and *tetB*(P)	L20800
*tet*(Q)	Z21523
*tet*(S)	FN555436
*tet*(T)	L42544
*tet*(W)	AJ222769
*tet*(32)	AJ295238
*tet*(36)	AJ514254
*tet*(44)	FN594949
*tet*(61)	KY887560
*otr*(A)	X53401
*tet*	M74049

The list is adapted from http://faculty.washington.edu/marilynr/tetweb4.pdf.

To reconstruct a global phylogeny, an additional collection of clonal complex (CC) 230 isolates (*n*=130) from previous studies,^16–20^ together with the CC230 collection (*n*=259) in the GPS dataset were included. The phylogeny was built as previously described.^14^ Based on the international genomic definition of pneumococcal lineages, all CC230 isolates in this study belong to GPS Cluster (GPSC)10.^14^ The metadata and analysis results of CC230 can be interactively visualized online using the Microreact tool at https://microreact.org/project/GPS_tetSM.

### Temporal changes of tet(S/M) CC230 sublineage

Coalescent analysis was performed on *tet*(S/M) CC230 sublineage (*n*=129) to date the most recent common ancestor (MRCA) and reconstruct the population demographic history. First, we tested the presence of temporal signal by a linear regression of root-to-tip distances against year of collection using TempEst v1.5.^21^ Next, a timed phylogeny was constructed using BEAST v2.4.1.^22^ The Markov chain Monte Carlo (MCMC) chain was run for 100 million generations, sampled every 1000 states using the general time-reversible (GTR) model of nucleotide substitution and the discrete gamma model of heterogeneity among sites. Finally, the population demographic history was reconstructed using a birth–death model^23^ to examine the temporal changes with the *tet*(S/M) CC230 sublineage invasive isolates (*n*=105) but not carriage isolates (*n*=24), because the model assumes that once an individual is diagnosed with IPD, the individual is no longer transmitting due to treatment and recovery, death or being socially removed from susceptible individuals. Thus, it appeared to be logical to apply this model to the disease but not carriage isolates. This model overcomes the limitations of the coalescent-based skyline plot and is able to examine whether introduction of an intervention had an impact on the epidemiological dynamics in a bacterial population.^24^ The birth–death skyline plot shows the effective reproductive number (R) over time. R is defined as the number of expected secondary infections from an infected individual. R>1 indicates a growing epidemic, whereas R<1 indicates a declining epidemic. Notably, R ≥ 1 can be reflected in the coalescent-based skyline plot analysis, whereas R<1 cannot. Therefore, we expected the birth–death skyline model would be a better fit for our data. Other Bayesian population-size models (coalescent constant, coalescent exponential and Bayesian skyline), in combination with strict and lognormal-relaxed molecular clocks, were also applied for comparisons using BEAST.

### Integrative and conjugative element (ICE)

The ICE was extracted from the *de novo* assemblies of CC230 isolates and compared using EasyFig version 2.2.2. The NCBI accession numbers for the representative ICE sequences in Figure 5 are FM211187 (ICESp23FST81), MH283017 [ICE*Sp*14ST230 with *tet*(M)], MH283012 [ICE*Sp*14ST230 with *tet*(S/M) and omega cassettes], MH283013 [ICE*Sp*14ST230 with *tet*(M) and omega], MH283012 [ICE*Sp*14ST230 with *tet*(M) and Tn*917*], MH283016 (ICE*Sp*19AST2013), MH283015 (ICE*Sp*17FST8812) and MH283014 (ICE*Sp*14ST156).

## Results

### Prevalence of tet(S/M) in a global collection of S. pneumoniae

A tetracycline resistance gene, *tet*(S/M), was identified in 131 pneumococcal isolates (1%, 131/12 254) from South Africa (*n*=123), Malawi (*n*=5), Brazil (*n*=1), Mozambique (*n*=1) and the USA (*n*=1). They were isolated from sterile body sites (invasive isolates) [blood (*n*=73), CSF (*n*=30) and pleural fluid (*n*=4)] and from the nasopharynx (carriage isolates) (*n*=24). In South Africa, *tet*(S/M) was found in 3.5% (103/2920) of the invasive isolates that were submitted to the GPS project from 2005 to 2014 and 1.2% (20/1701) of the carriage isolates that were collected in Agincourt and Soweto between 2009 and 2013. Of the 103 invasive isolates, 94% (97/103) were from children with IPD aged ≤5 years (Figure S1). HIV status was known in only 44% (54/123) of individuals with *tet*(S/M)-positive pneumococci; 59% (32/54) were HIV positive, of which 94% (30/32) were children aged ≤5 years.

**Figure 1.**
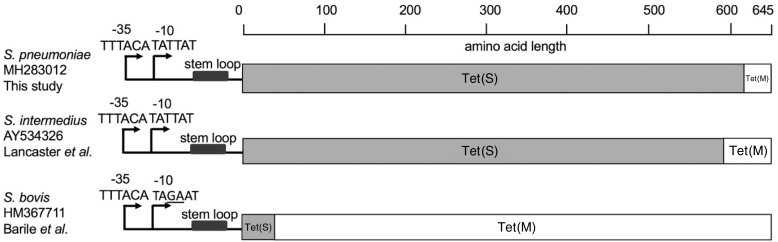
Schematic representation of the mosaic structure of *tet*(S/M) alleles of the current and previous studies. The bars in grey and in white indicate amino acid sequences with high identity to Tet(S) and Tet(M), respectively. The reference sequences for *tet*(M) and *tet*(S) were retrieved from NCBI GenBank using accession numbers M85225 and FN555436, respectively.

MIC of tetracycline was determined for 121 *tet*(S/M)-positive isolates by either Etest (*n*=73) or broth dilution (*n*=48). The remaining 10 isolates were either non-viable (*n*=5) or unable to be tested due to inadequate resources (*n*=5). The median MIC was 8 mg/L (IQR 6–8) by Etest and 16 mg/L by broth dilution. Based on the CLSI guideline, 99% (120/121) and 1% (1/121) were fully (≥4 mg/L) and intermediately (2–3 mg/L) resistant to tetracycline, respectively. The *tet*(S/M) in this study showed 100% nucleotide identity, except for one isolate (GPS_ZA_1982) from South Africa, which varied from the others (G1769A) and resulted in the substitution R590Q. This isolate remained resistant to tetracycline with an MIC of >8 mg/L when measured by broth dilution. Unlike the two previously reported *tet*(S/M) alleles from *S. intermedius*^11^ and *S. bovis*,^12^ the amino acid sequence of Tet(S/M) in this study showed 100% identity to Tet(S) (NCBI accession number FN555436) across the first 613 amino acids, with the final 32 amino acids at the C-terminus end being identical to Tet(M) (NCBI accession number M85225) (Figure 1). The promoter region was intact in all *tet*(S/M)-positive isolates and was of *tet*(M) origin, rather than *tet*(S) origin. Between the promoter region and the start codon of *tet*(S/M), a 38 bp stem loop, which is potentially involved in transcriptional regulation,^25^ was found in all *tet*(S/M) genes (Figure 1), apart from one disease isolate (GPS_ZA_1926) from South Africa. The deletion did not affect the tetracycline resistance level, as the MIC remained at >8 mg/L when measured by the broth dilution method. No other *tet* genes (Table 1) were detected in any *tet*(S/M)-positive isolates, strongly indicating that Tet(S/M) conferred resistance to tetracycline in *S. pneumoniae*.

### Phylogeny and characteristics of the tet(S/M) CC230 sublineage

**Figure 2.**
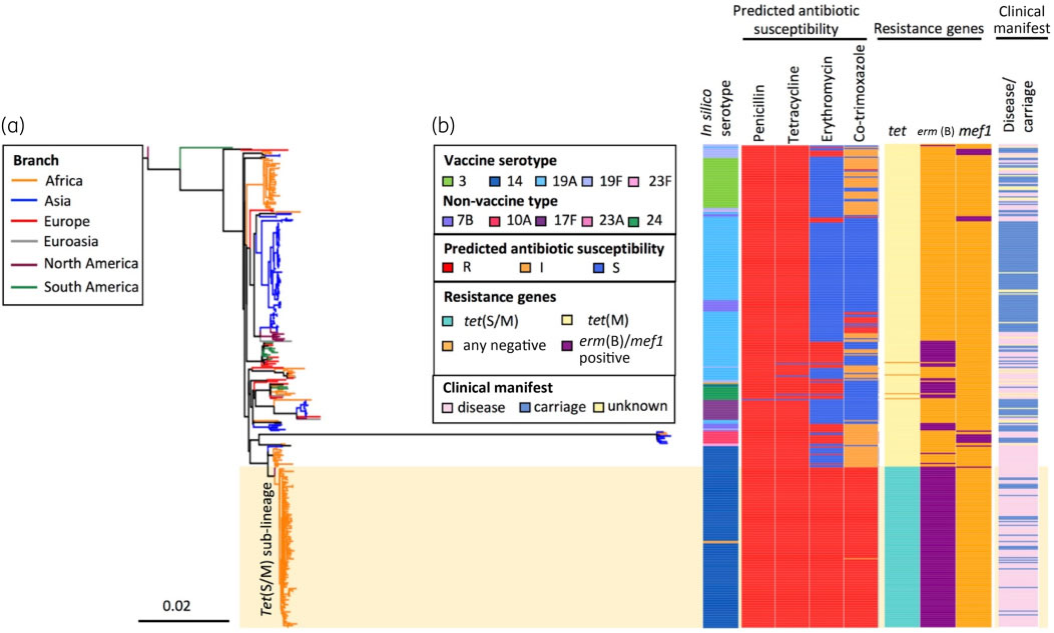
An SNP tree constructed with CC230 *tet*(S/M)-positive isolates (*n*=129) and *tet*(S/M)-negative carriage/disease isolates (*n*=260) collected from 20 countries. The tree was built based on 13 405 SNPs extracted from an alignment outside recombination regions, created by mapping reads of each isolate to the sequence of an ST230 reference strain, PMEN global clone Denmark^14^-32, PMEN32 (ENA accession number ERS1706837). Penicillin resistance was predicted based on the *pbp1a*, *pbp2x* and *pbp2b* sequences;^38^^,^^39^ tetracycline and erythromycin resistance were predicted based on the presence of *tet*(M), *tet*(O) and *tet*(S/M), and *erm*(B) and *mef*(A), respectively. Co-trimoxazole resistance was predicted based on the presence of mutation I100L in *folA* and any indel within amino acid residues 56–67 in *folP*, while the presence of either mutation was predicted to confer a co-trimoxazole-intermediate phenotype.

Of the 131 *tet*(S/M)-positive isolates, 129 belonged to CC230; one Brazilian and one Malawian isolate belonged to CC156 and ST5359 (a singleton not belonging to any CC), respectively. The global CC230 phylogeny showed that all *tet*(S/M)-positive isolates formed a sublineage predicted to be resistant to penicillin, erythromycin, tetracycline and co-trimoxazole (Figure 2). The *tet*(S/M) sublineage was associated predominantly with VT 14 (98%, 127/129) but was also found in two NVT 23A isolates. The two serotype 23A isolates, which both belonged to ST11106 (a single-locus variant of ST230), were recovered from infants after the introduction of PCV13. One was isolated from a nasopharyngeal sample in Soweto in 2012 and the other from blood culture in Johannesburg in 2014. The serotype 23A *cps* locus sequences of these two isolates were identical and their *cps*-flanking *pbp* loci (*pbp1a* and *pbp2x*) were also identical to the majority of the serotype 14 isolates within the *tet*(S/M) sublineage, exhibiting resistance to penicillin with an MIC of 2 mg/L. To identify the potential donor of the serotype 23A *cps* locus, a phylogenetic tree was built using the *cps* sequences from all serotype 23A (*n*=130, belong to eight lineages) pneumococci in the GPS database. This analysis showed that the serotype 23A *cps* loci of these two CC230 isolates clustered with those originating from a serogroup 23 lineage GPSC7, which is predominantly (99%, 145/146) represented by CC439 (Figure S2), with pairwise nucleotide similarity of 99.97% (24 818/24 825) and 100% coverage. The seven nucleotide variations were found within the IS*630* transposase downstream of *dexB*.

### Temporal spread of the tet(S/M) CC230 sublineage

**Figure 3.**
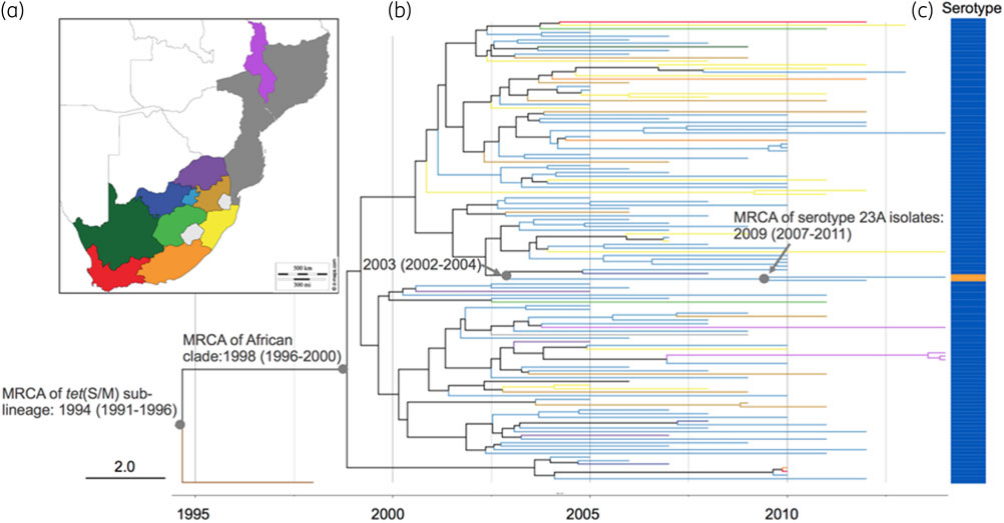
(a) Malawi, Mozambique and administration regions of South Africa. (b) Timed phylogeny for *S. pneumoniae tet*(S/M) CC230 sublineage (*n*=129) reconstructed using BEAST. Tree branches are coloured according to the geographical locations in (a), except for the branch for an isolate collected from the USA, coloured in brown. (c) Vaccine serotype 14 is indicated in blue, whereas non-vaccine serotype 23A is indicated in orange.

**Figure 4.**
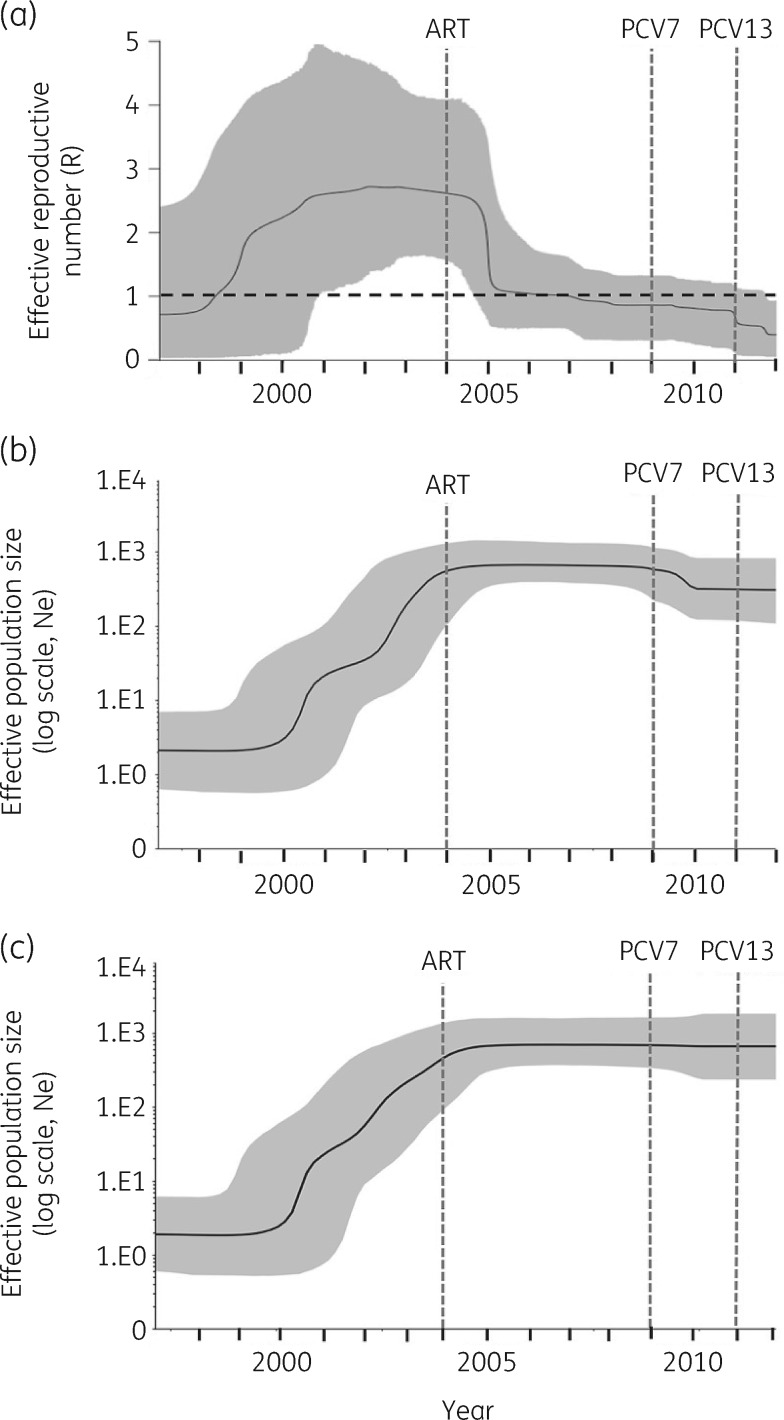
(a) Birth–death skyline plot of inferred changes in R of *S. pneumoniae tet*(S/M) CC230 sublineage using IPD isolates (*n*=105). (b) Coalescent-based skyline plot of inferred changes in the effective population size (Ne) of the *S. pneumoniae tet*(S/M) CC230 sublineage using both IPD and carriage isolates (*n*=129) and (c) using only IPD isolates (*n*=105). The black continuous line shows the median of R in (a) and Ne in (b) and (c). The background area represents the 95% HPD intervals. R>1 indicates a growing epidemic, whereas R<1 indicates a declining epidemic.

The sublineage showed a temporal signal in terms of SNP accumulation against time (*R*^2^* *=* *0.4094, *P*=0.001; Figure S3). Using a birth–death model in BEAST, the *tet*(S/M) sublineage was estimated to emerge around 1994 [95% highest posterior density (HPD): 1991–96]; the MRCA for the African clade was 1998 (95% HPD: 1996–2000) and for the two serotype 23A isolates was 2009 (95% HPD: 2007–11) (Figure 3). The temporal changes of spread were reconstructed based on a birth–death skyline plot and a coalescent-based skyline plot (Figure 4). Both skyline plots showed that the *tet*(S/M) sublineage expanded at the beginning of the year 2000 and growth continued until around 2004. The decline of the *tet*(S/M) sublineage was only captured by the birth–death skyline plot in or around 2005, from expected secondary infections (R) of ∼2.5 to ∼1, and steadily declined until 2012 when the median and HPD of R were <1, indicating a declining epidemic. The coalescent-based skyline plot failed to detect the impact of the epidemic decline, as described in a previous study.^23^

### ICE carrying tet(S/M)

**Figure 5.**
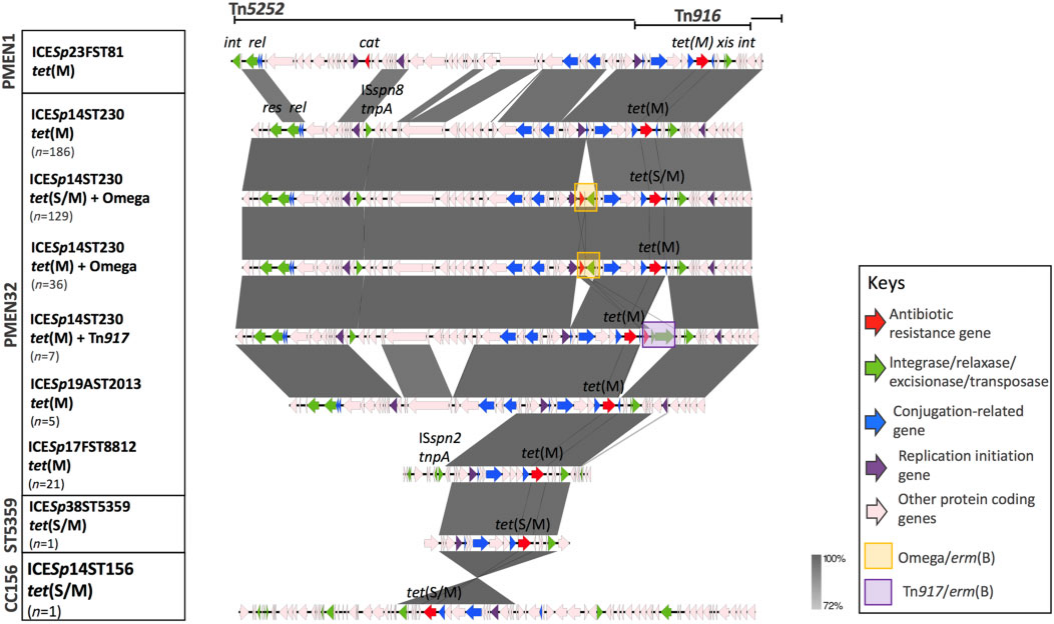
Comparison of ICE identified in CC230 with Spain23^F^-1 (PMEN1). Grey bands between the sequences indicate BLASTN matches.

The acquisition of tetracycline and erythromycin resistance determinants by CC230 was the result of the insertion of a Tn*5253*-type ICE, which shared a similar structure to ICE*Sp*23FST81 identified in PMEN1 (Figure 5). Both the *tet*(M) (*n*=255) and *tet*(S/M) (*n*=131) genes detected in this study were carried on a conserved conjugative Tn*916* transposon (Figure S4). Of the 172 macrolide-resistant isolates, insertions of either the ‘omega’ element (*n*=165) or Tn*917* (*n*=7) harbouring *erm*(B) were found upstream or downstream of the *tet* gene, respectively (Figure 5). The insertion of the ‘omega’ element truncated the gene encoding the replication initiation factor, creating an 8 bp DR, CAAAAAAA. The insertion of Tn*917* disrupted the gene *orf9*, which encodes a putative conjugative transposon regulator; no DRs were found.

## Discussion

We used WGS to identify a novel mosaic structure of *tet*(S/M) in *S. pneumoniae*. This approach overcame the limitation of PCR that requires specific primers to detect known antibiotic resistance genes. Compared with *tet*(M), the prevalence of *tet*(S/M) was low. They were mainly found in a CC230 sublineage that predominantly expressed VT 14 and exhibited MDR in South Africa. Together with its conserved nucleotide sequence and genomic location, our finding strongly suggested a clonal expansion of *tet*(S/M)-positive CC230 isolates within South Africa prior to the introduction of PCVs.

Unlike what was observed in other countries, CC230 in South Africa predominantly expressed a highly invasive serotype 14 capsule^14^ and was the clone that represented most of the serotype 14 isolates (43%) in the pre-vaccine era, when serotype 14 was the most prevalent serotype causing IPD in South Africa.^26^ Any controlling measure to decrease this lineage would not only result in a reduction in the IPD burden but also MDR IPD incidence.

The birth–death model estimated that the decline of the *tet*(S/M) CC230 sublineage started around 2005, one year after the national ART programme was launched to treat HIV-infected individuals in South Africa.^27^ The prediction was consistent with a 41% reduction of IPD incidence among HIV-infected children after the introduction of ART.^27^ Among the IPD caused by *tet*(S/M) CC230 isolates, almost 60% occurred in HIV-positive children. This observational evidence strengthened the suggestion that ART was likely to contribute to the decline before PCV introduction. In contrast, the large-scale use of co-trimoxazole as prophylaxis to prevent bacterial infections among HIV-positive individuals was unlikely to be responsible for the decline, as the sublineage was resistant to co-trimoxazole. The further decline in 2012 predicted by the model echoed the epidemiological finding that IPD caused by VT pneumococci significantly decreased among children in 2012.^28^ Our finding demonstrated that we could effectively reconstruct the temporal spread of an epidemic using genomic data and highlighted the possible use of routine genomic surveillance to model outbreaks as they occur.

The MRCA of two CC230 isolates expressing NVT 23A was dated to emerge around 2009, the year when PCV7 was introduced. However, the long branch leading to the MRCA from the internal node that was shared with the closely related serotype 14 isolates indicated that the window of time for capsular switching could be between 2002 and 2011. Although the invasive disease potential for serotype 23A is low,^14^ a significant increase of this serotype in IPD cases was reported from England,^29^ Stockholm^30^ and Taiwan^31^ after the implementation of PCV13. Serotype 23A is primarily associated with CC338 (GPSC5, PMEN26) and CC439 (GPSC7) and is thus rarely found in a CC230 genetic background. Such serotype and genotype combinations were only identified in two ST9396 isolates (single-locus variant of ST230) from China in 2013 and one ST10921 isolate (double-locus variant of ST230) from Poland in 2013 in the MLST database. In South Africa, CC439 accounted for 62% of serotype 23A (both carriage and disease) isolates^14^ and is the potential donor of the serotype 23A *cps* to the *tet*(S/M) CC230 sublineage, highlighting that capsular switching with the prevalent NVT lineage could enable a VT lineage to evade the vaccine. Capsular switching is usually a result of homologous recombination. When compared with 620 other GPSCs, GPSC10, including 98% (258/262) of CC230 isolates, is a very recombinogenic lineage, which had a significantly high recombination rate [ratio of base substitutions predicted to have been imported through recombination to those occurring through point mutation for GPSC10: 10.9 versus median of 35 dominant GPSCs: 8.3 (IQR, 5.7–10.7), *P*<0.0001, Wilcoxon signed-rank test].^14^ Given this recombinogenic nature, together with the established MDR genotypes, it is of concern that any further capsular switching may increase the chance of this MDR lineage surviving and continuing to cause invasive disease.

Like *tet*(M), *tet*(S/M) was also carried by Tn*916*, which was reported as a mobile conjugative transposon with a broad host range.^11^ The conserved genetic environment of *tet*(M) and *tet*(S/M) indicates that the recombination resulting in the mosaic structure of *tet*(S/M) probably occurred after the acquisition of the gene by Tn*916*. Comparison of *tet*(M) sequences in the current collection also revealed a high degree of allelic variations that were probably due to homologous recombination.^32^ This finding is consistent with previous studies that suggested that *tet* evolved separately from Tn*916.*^6^^,^^32^ However, the driving force behind the evolution of *tet* genes remains unclear, given that tetracycline was not used as a first-line antibiotic to treat pneumococcal disease and was seldom used in young children.^33^ The allelic diversity of *tet* may be maintained by: (i) frequent recombination among *S. pneumoniae* and with closely related species such as normal nasopharyngeal resident *Streptococcus mitis*^34^ and the zoonotic pathogen *S. suis*;^35^ and (ii) antibiotic-selective pressure via the food chain, as tetracycline is widely used in agriculture^36^ and its residue is detected in milk.^37^ Future studies that investigate the driving force behind the evolution of *tet* will improve our understanding to develop preventive measures to reduce tetracycline resistance in *S. pneumoniae*.

In conclusion, we identified the tetracycline-resistance determinant *tet*(S/M) in *S. pneumoniae* and showed that its dissemination is due to a clonal expansion of the MDR lineage CC230 in South Africa, where the HIV burden is high. With genomic data, we successfully detected the decline in transmission of this MDR lineage using a birth–death model and the fall of this lineage may correlate with the improved treatment of HIV-infected individuals and the implementation of PCVs. Capsular switching within this lineage is potentially of public health importance and may erode the beneficial effect brought about by the implementation of PCVs. The capacity for continuous genomic surveillance in the post-vaccine era provides critical opportunities for monitoring and forecasting the rise of MDR pneumococcal lineages that may also undergo vaccine evasion through capsular switching events.

## Supplementary Material

dkz477_Supplementary_Data

## References

[1] Lo SW, Gladstone RA, van Tonder AJ et al Pneumococcal lineages associated with serotype replacement and antibiotic resistance in childhood invasive pneumococcal disease in the post-PCV13 era: an international whole-genome sequencing study. Lancet Infect Dis 2019; 19: 759–69. 31196809 10.1016/S1473-3099(19)30297-X PMC7641901

[2] Wyres KL, Lambertsen LM, Croucher NJ et al Pneumococcal capsular switching: a historical perspective. J Infect Dis 2013; 207: 439–49. 23175765 10.1093/infdis/jis703 PMC3537446

[3] Metcalf BJ, Gertz RE Jr, Gladstone RA et al Strain features and distributions in pneumococci from children with invasive disease before and after 13-valent conjugate vaccine implementation in the USA. Clin Microbiol Infect 2016; 22: 60.e9–29. 10.1016/j.cmi.2015.08.027 PMC4721534 26363404

[4] Chochua S, Metcalf BJ, Li Z et al Invasive serotype 35B pneumococci including an expanding serotype switch lineage, United States, 2015-2016. Emerg Infect Dis 2017; 23: 922–30. 28516866 10.3201/eid2306.170071 PMC5443455

[5] Olarte L, Kaplan SL, Barson WJ et al Emergence of multidrug-resistant pneumococcal serotype 35B among children in the United States. J Clin Microbiol 2017; 55: 724–34. 27847379 10.1128/JCM.01778-16 PMC5328440

[6] Wyres KL, van Tonder A, Lambertsen LM et al Evidence of antimicrobial resistance-conferring genetic elements among pneumococci isolated prior to 1974. BMC Genomics 2013; 14: 500. 23879707 10.1186/1471-2164-14-500 PMC3726389

[7] Widdowson CA, Klugman KP, Hanslo D. Identification of the tetracycline resistance gene, *tet*(O), in *Streptococcus pneumoniae*. Antimicrob Agents Chemother 1996; 40: 2891–3. 9124862 10.1128/aac.40.12.2891 PMC163643

[8] Charpentier E, Gerbaud G, Courvalin P. Characterization of a new class of tetracycline-resistance gene *tet*(S) in *Listeria monocytogenes* BM4210. Gene 1993; 131: 27–34. 8370538 10.1016/0378-1119(93)90665-p

[9] Huang J, Ma J, Shang K et al Evolution and diversity of the antimicrobial resistance associated mobilome in *Streptococcus suis*: a probable mobile genetic elements reservoir for other streptococci. Front Cell Infect Microbiol 2016; 6: 118. 27774436 10.3389/fcimb.2016.00118 PMC5053989

[10] Liu LC, Tsai JC, Hsueh PR et al Identification of *tet*(S) gene area in tetracycline-resistant *Streptococcus dysgalactiae* subsp. *equisimilis* clinical isolates. J Antimicrob Chemother 2008; 61: 453–5. 18156606 10.1093/jac/dkm500

[11] Lancaster H, Roberts AP, Bedi R et al Characterization of Tn*916*S, a Tn*916*-like element containing the tetracycline resistance determinant *tet*(S). J Bacteriol 2004; 186: 4395–8. 15205444 10.1128/JB.186.13.4395-4398.2004 PMC421593

[12] Barile S, Devirgiliis C, Perozzi G. Molecular characterization of a novel mosaic *tet*(S/M) gene encoding tetracycline resistance in foodborne strains of *Streptococcus bovis*. Microbiology 2012; 158: 2353–62. 22723288 10.1099/mic.0.058206-0 PMC3542815

[13] CLSI. *Performance Standards for Antimicrobial Susceptibility Testing—Twenty-Fourth Edition: M100* 2014.

[14] Gladstone RA, Lo SW, Lees JA et al International genomic definition of pneumococcal lineages, to contextualise disease, antibiotic resistance and vaccine impact. EBioMedicine 2019; 43: 338–46. 31003929 10.1016/j.ebiom.2019.04.021 PMC6557916

[15] Hunt M, Mather AE, Sanchez-Buso L et al ARIBA: rapid antimicrobial resistance genotyping directly from sequencing reads. Microb Genom 2017; 3: e000131. 29177089 10.1099/mgen.0.000131 PMC5695208

[16] Chewapreecha C, Harris SR, Croucher NJ et al Dense genomic sampling identifies highways of pneumococcal recombination. Nat Genet 2014; 46: 305–9. 24509479 10.1038/ng.2895 PMC3970364

[17] Quirk SJ, Haraldsson G, Erlendsdottir H et al Effect of vaccination on pneumococci isolated from the nasopharynx of healthy children and the middle ear of children with otitis media in Iceland. J Clin Microbiol 2018; 56: e01046-18. 30257906 10.1128/JCM.01046-18 PMC6258863

[18] Chaguza C, Cornick JE, Andam CP et al Population genetic structure, antibiotic resistance, capsule switching and evolution of invasive pneumococci before conjugate vaccination in Malawi. Vaccine 2017; 35: 4594–602. 28711389 10.1016/j.vaccine.2017.07.009 PMC5571440

[19] Cremers AJ, Mobegi FM, de Jonge MI et al The post-vaccine microevolution of invasive *Streptococcus pneumoniae*. Sci Rep 2015; 5: 14952. 26492862 10.1038/srep14952 PMC4615977

[20] Gladstone RA, Devine V, Jones J et al Pre-vaccine serotype composition within a lineage signposts its serotype replacement – a carriage study over 7 years following pneumococcal conjugate vaccine use in the UK. Microbial Genomics 2017; 3: e000119. 29026652 10.1099/mgen.0.000119 PMC5628697

[21] Rambaut A, Lam TT, Max Carvalho L et al Exploring the temporal structure of heterochronous sequences using TempEst (formerly Path-O-Gen). Virus Evol 2016; 2: vew007. 27774300 10.1093/ve/vew007 PMC4989882

[22] Bouckaert R, Heled J, Kuhnert D et al BEAST 2: a software platform for Bayesian evolutionary analysis. PLoS Comput Biol 2014; 10: e1003537. 24722319 10.1371/journal.pcbi.1003537 PMC3985171

[23] Stadler T, Kuhnert D, Bonhoeffer S et al Birth–death skyline plot reveals temporal changes of epidemic spread in HIV and hepatitis C virus (HCV). Proc Natl Acad Sci USA 2013; 110: 228–33. 23248286 10.1073/pnas.1207965110 PMC3538216

[24] Kuhnert D, Coscolla M, Brites D et al Tuberculosis outbreak investigation using phylodynamic analysis. Epidemics 2018; 25: 47–53. 29880306 10.1016/j.epidem.2018.05.004 PMC6227250

[25] Chopra I, Roberts M. Tetracycline antibiotics: mode of action, applications, molecular biology, and epidemiology of bacterial resistance. Microbiol Mol Biol Rev 2001; 65: 232–60. 11381101 10.1128/MMBR.65.2.232-260.2001 PMC99026

[26] Ndlangisa KM, du Plessis M, Wolter N et al Population snapshot of *Streptococcus pneumoniae* causing invasive disease in South Africa prior to introduction of pneumococcal conjugate vaccines. PLoS One 2014; 9: e107666. 25233455 10.1371/journal.pone.0107666 PMC4169438

[27] Nunes MC, von Gottberg A, de Gouveia L et al The impact of antiretroviral treatment on the burden of invasive pneumococcal disease in South African children: a time series analysis. AIDS 2011; 25: 453–62. 21178754 10.1097/QAD.0b013e328341b7f1

[28] von Gottberg A, de Gouveia L, Tempia S et al Effects of vaccination on invasive pneumococcal disease in South Africa. N Engl J Med 2014; 371: 1889–99. 25386897 10.1056/NEJMoa1401914

[29] Moore CE, Paul J, Foster D et al Reduction of invasive pneumococcal disease 3 years after the introduction of the 13-valent conjugate vaccine in the Oxfordshire region of England. J Infect Dis 2014; 210: 1001–11. 24719477 10.1093/infdis/jiu213

[30] Galanis I, Lindstrand A, Darenberg J et al Effects of PCV7 and PCV13 on invasive pneumococcal disease and carriage in Stockholm, Sweden. Eur Respir J 2016; 47: 1208–18. 26797033 10.1183/13993003.01451-2015 PMC4819883

[31] Su LH, Kuo AJ, Chia JH et al Evolving pneumococcal serotypes and sequence types in relation to high antibiotic stress and conditional pneumococcal immunization. Sci Rep 2015; 5: 15843. 26522920 10.1038/srep15843 PMC4629140

[32] Oggioni MR, Dowson CG, Smith JM et al The tetracycline resistance gene *tet*(M) exhibits mosaic structure. Plasmid 1996; 35: 156–63. 8812782 10.1006/plas.1996.0018

[33] WHO. Scientific basis of WHO recommendations for treatment of pneumonia. In: *Revised WHO Classification and Treatment of Pneumonia in Children at Health Facilities: Evidence Summaries* 2014 https://www.ncbi.nlm.nih.gov/books/NBK264159/. 25535631

[34] Kilian M, Riley DR, Jensen A et al Parallel evolution of *Streptococcus pneumoniae* and *Streptococcus mitis* to pathogenic and mutualistic lifestyles. MBio 2014; 5: e01490-14. 25053789 10.1128/mBio.01490-14 PMC4120201

[35] Ye C, Bai X, Zhang J et al Spread of *Streptococcus suis* sequence type 7, China. Emerg Infect Dis 2008; 14: 787–91. 18439362 10.3201/eid1405.070437 PMC2600270

[36] Granados-Chinchilla F, Rodriguez C. Tetracyclines in food and feedingstuffs: from regulation to analytical methods, bacterial resistance, and environmental and health implications. J Anal Methods Chem 2017; 2017: 1315497. 28168081 10.1155/2017/1315497 PMC5266830

[37] de Albuquerque Fernandes SA, Magnavita AP, Ferrao SP et al Daily ingestion of tetracycline residue present in pasteurized milk: a public health problem. Environ Sci Pollut Res Int 2014; 21: 3427–34. 24243094 10.1007/s11356-013-2286-5

[38] Li Y, Metcalf BJ, Chochua S et al Penicillin-binding protein transpeptidase signatures for tracking and predicting β-lactam resistance levels in *Streptococcus pneumoniae*. MBio 2016; 7: e00756-16. 27302760 10.1128/mBio.00756-16 PMC4916381

[39] Li Y, Metcalf BJ, Chochua S et al Validation of β-lactam minimum inhibitory concentration predictions for pneumococcal isolates with newly encountered penicillin binding protein (PBP) sequences. BMC Genomics 2017; 18: 621. 28810827 10.1186/s12864-017-4017-7 PMC5558719

