# Supplementary material for: A mosaic tetracycline resistance gene tet(S/M) detected in an MDR pneumococcal CC230 lineage that underwent capsular switching in South Africa
Source: J Antimicrob Chemother. 2019 Dec 2;75(3):512–20. doi: 10.1093/jac/dkz477 (PMC7021099; doi:10.1093/jac/dkz477)
Supplement: dkz477_Supplementary_Data [file dkz477_supplementary_data.zip › Supplementary_Figures S1 to S4.docx]

**Supplementary data**

**Figure S1.** Number of *tet*(S/M)-positive isolates, by age and clinical manifest. Bars in pink indicated invasive pneumococcal disease isolates while blue indicated carriage isolates. Solid and hatched bars indicated isolates collected from South Africa and elsewhere, respectively.


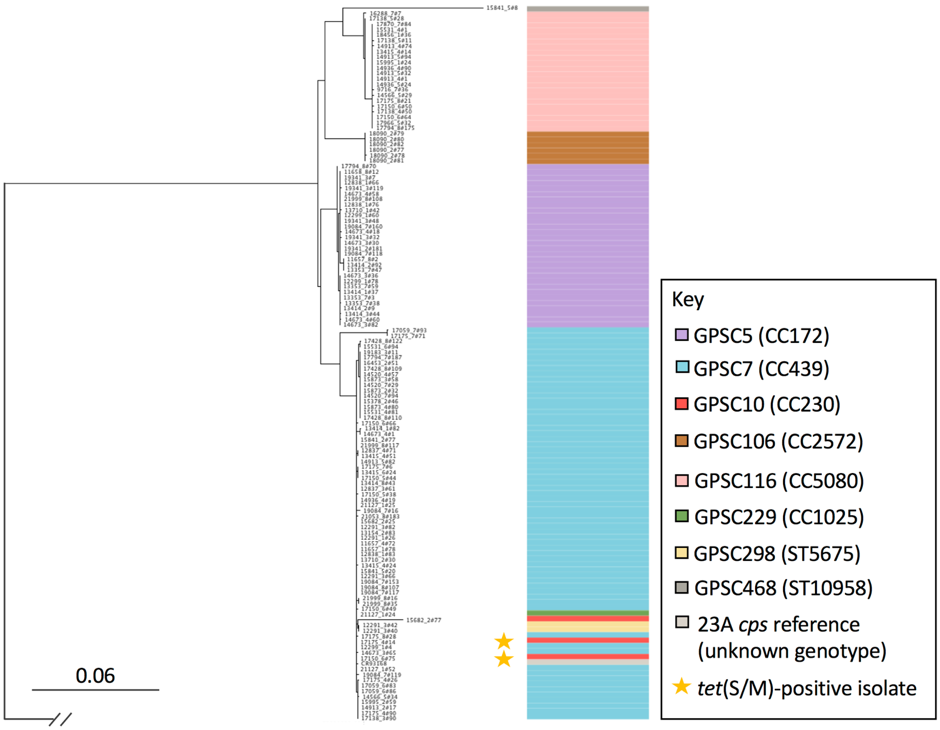


**Figure S2.** Maximum likelihood phylogenetic tree was constructed using 2,178 SNPs extracted from a 21,296-bp alignment of serotype 23A *cps* locus sequences from the serotype 23A S. pneumoniae isolates (n=130) in the GPS curated dataset. This analysis used the serotype 23F *cps* locus reference sequence (accession number CR931685) as the outgroup on which to root the tree. The serotype 23A *cps* reference sequence (accession number CR931683) was included. The primary clonal complex (CC) or sequence type (ST) associated with Global Pneumococcal Sequence Cluster (GPSC) was indicated in parentheses.


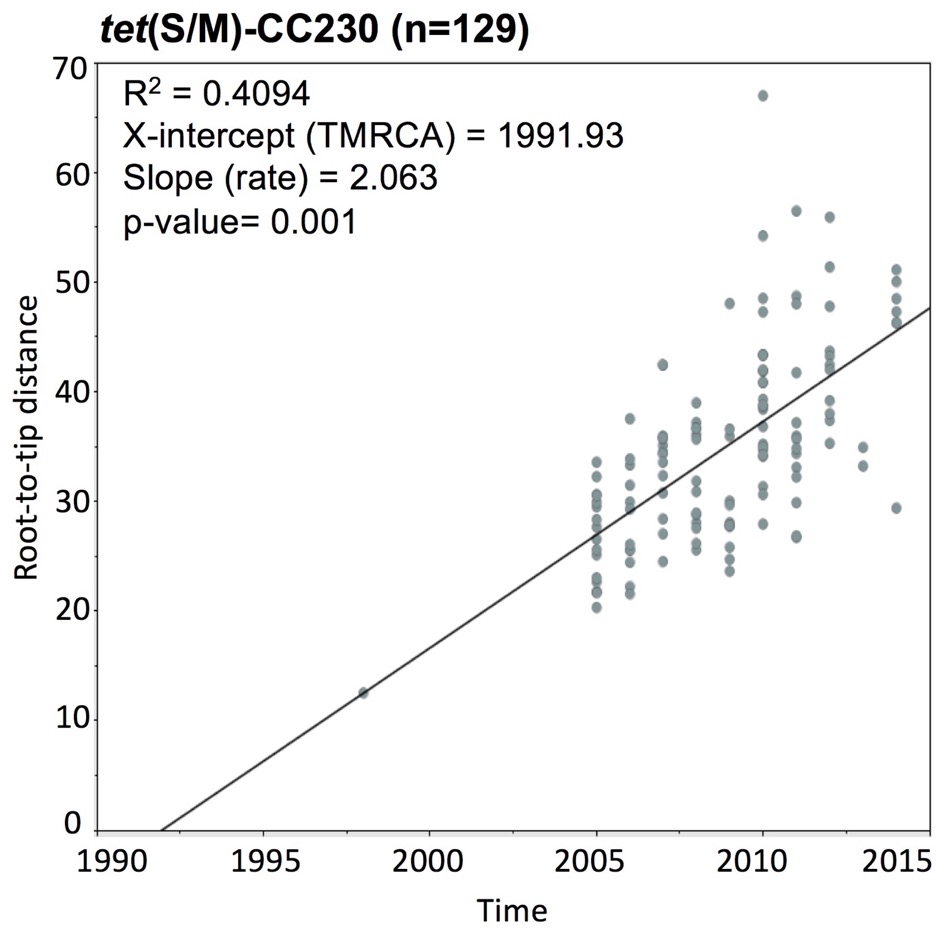


**Figure S3.** Linear regression of root-to-tip distance against time on *tet*(S/M)-CC230 lineage (n=129) using TempEST v1.5. TempEst detected a significant positive correlation of year of collection with its genetic distance from the root, indicating a signal of a ‘molecular clock’, with which isolates measurably diversifying from their last common ancestor over time.


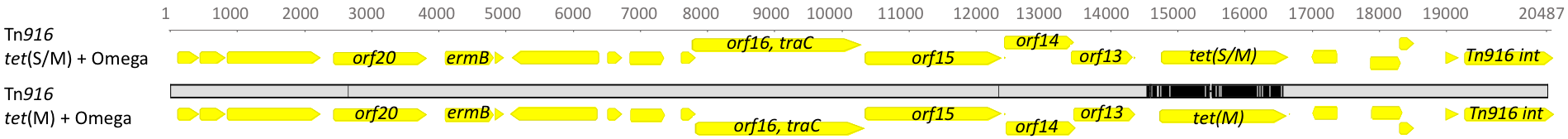


**Figure S4.** Comparison of Tn*916* carrying *tet*(S/M) and *tet*(M) in clonal complex (CC)230. The yellow arrows indicated protein coding region. The grey band between the sequence indicates BLASTN match and black vertical lines shows the unmatched nucleotide bases.
